# 2-Chloro-3-(4-chloro­benzamido)-1,4-naphthoquinone

**DOI:** 10.1107/S1600536808040993

**Published:** 2008-12-10

**Authors:** Yakini Brandy, Ray J. Butcher, Tolulope A. Adesiyun, Solomon Berhe, Oladapo Bakare

**Affiliations:** a Department of Chemistry, Howard University, 525 College Street NW, Washington, DC 20059, USA

## Abstract

The naphthoquinone ring is almost perpendicular [dihedral angle 71.02 (3)°] to the phenyl group of the title compound, C_17_H_9_Cl_2_NO_3_, while the dihedral angle between the amide group and the 4-chloro­phenyl ring is 21.9 (2)°. The conformation of the N—H and C=O bonds are *anti* to each other. N—H⋯Cl hydrogen bonds link the mol­ecules into chains in the *a*-axis direction. In addition, these chains are linked by weak inter­molecular C—H⋯O inter­actions.

## Related literature

For similar structures see: Lien *et al.* (1997 ▶); Huang *et al.* (2005 ▶); Bakare *et al*. (2003 ▶); Copeland *et al.* (2007 ▶); Win *et al.* (2005 ▶); Rubin-Preminger *et al.* (2004 ▶). For related literature, see: Gowda, Kožíšek *et al.* (2008 ▶); Gowda, Tokarčík *et al.* (2008 ▶); van Oosten *et al.* (2008 ▶); Shen *et al.* (2008 ▶).
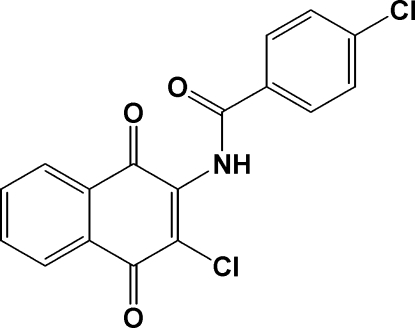


## Experimental

### 

#### Crystal data

C_17_H_9_Cl_2_NO_3_
*M* *_r_* = 346.15
Monoclinic, 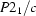

*a* = 5.6011 (2) Å
*b* = 8.7237 (3) Å
*c* = 29.7957 (9) Å
β = 93.504 (3)°
*V* = 1453.16 (8) Å^3^
*Z* = 4
Mo *K*α radiation
μ = 0.46 mm^−1^
*T* = 200 (2) K
0.49 × 0.41 × 0.12 mm

#### Data collection

Oxford Diffraction Gemini R diffractometer
Absorption correction: multi-scan (*CrysAlis RED*; Oxford Diffraction, 2007 ▶) *T*_min_ = 0.887, *T*_max_ = 1.000 (expected range = 0.839–0.946)
13882 measured reflections
4842 independent reflections
2832 reflections with *I* > 2σ(*I*)
*R*_int_ = 0.035

#### Refinement

*R*[*F* ^2^ > 2σ(*F* ^2^)] = 0.038
*wR*(*F* ^2^) = 0.086
*S* = 0.93
4842 reflections
208 parameters
H-atom parameters constrained
Δρ_max_ = 0.29 e Å^−3^
Δρ_min_ = −0.36 e Å^−3^

### 

Data collection: *CrysAlis CCD* (Oxford Diffraction, 2007 ▶); cell refinement: *CrysAlis RED* (Oxford Diffraction, 2007 ▶); data reduction: *CrysAlis RED*; program(s) used to solve structure: *SHELXS97* (Sheldrick, 2008 ▶); program(s) used to refine structure: *SHELXL97* (Sheldrick, 2008 ▶); molecular graphics: *SHELXTL* (Sheldrick, 2008 ▶); software used to prepare material for publication: *SHELXTL*.

## Supplementary Material

Crystal structure: contains datablocks global, I. DOI: 10.1107/S1600536808040993/at2690sup1.cif

Structure factors: contains datablocks I. DOI: 10.1107/S1600536808040993/at2690Isup2.hkl

Additional supplementary materials:  crystallographic information; 3D view; checkCIF report

## Figures and Tables

**Table 1:** Hydrogen-bond geometry (Å, °)

*D*—H⋯*A*	*D*—H	H⋯*A*	*D*⋯*A*	*D*—H⋯*A*
N—H0*A*⋯Cl1^i^	0.88	2.89	3.6491 (12)	145
C14—H14*A*⋯O2^ii^	0.95	2.40	3.2517 (19)	149

Symmetry codes: (i) 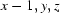; (ii) 
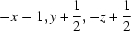
.

**Fig. 1.**
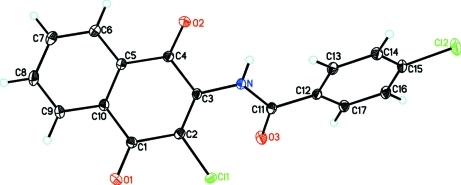
View of (I) showing the atom-labelling scheme. Displacement ellipsoids are drawn at the 20% probability level.

**Fig. 2.**
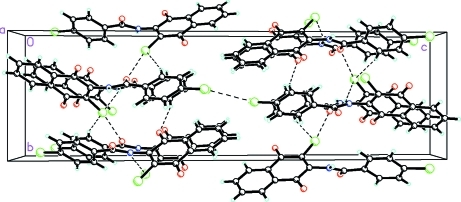
View of the packing viewed down the a axis. Dashed bonds show weak C—H···O interactions.

## supplementary crystallographic information

### Comment

The amido and imido derivatives of 3-chloro-1,4-naphthoquinone are well known for their anti-inflammatory, antiplatelet, antiallergic and anticancer activities (Lien *et al.*, 1997; Huang *et al.*, 2005; Bakare *et al.*, 2003; Copeland *et al.*, 2007). The title compound, 2-chloro-3-(*p*-chlorobenzamido)-1,4-naphthoquinone was obtained as an intermediate in the synthesis of some oxazolo-1,4-naphthoquinone and imido-substituted-1,4-naphthoquinone analogs.

The naphthoquinone ring is almost perpendicular to the phenyl group of the title compound C_17_H_9_Cl_2_NO_3_, while the dihedral angle betwen the amide group and the 4-chlorophenyl ring is 21.9 (2)° (Fig. 1). The conformation of the N—H and C=O bonds are anti to each other (Gowda, Kožíšek *et al.*, 2008; Gowda, Tokarčík *et al.*, 2008). N—H···Cl hydrogen bonds link the molecules into chains in the *a* direction. In addition, these chains are linked by weak intermolecular Ar—H···O interactions (Fig. 2, Table 1).

### Experimental

A mixture of 2-amino-3-chloro-1,4-naphthoquinone (213 mg, 1.03 mmol) and 4-chloro-benzoylchloride (2 ml) was refluxed for 2 1/2 h (powerstat setting at 70). The reaction mixture was cooled to room temperature. The precipitate was isolated by vacuum filtration and the yellow-grey solid was washed with diethyl ether. The crude was recrystallized from ethanol (20 ml) to obtain a yellow solid (67 mg, 18.8%). Crystals for *x*-ray study were obtained by recrystallization from methanol.

### Refinement

All H atoms were placed in geometrically idealized positions and constrained to ride on their parent atoms with C—H = 0.95 Å, N—H = 0.88 Å and *U*_iso_(H) = 1.2*U*_eq_(C, N).

### Crystal data

**Table:**

C_17_H_9_Cl_2_NO_3_	*F*(000) = 704
*M**_r_* = 346.15	*D*_x_ = 1.582 Mg m^−^^3^
Monoclinic, *P*2_1_/*c*	Mo *K*α radiation, λ = 0.71073 Å
Hall symbol: -P 2ybc	Cell parameters from 4629 reflections
*a* = 5.6011 (2) Å	θ = 4.6–32.5°
*b* = 8.7237 (3) Å	µ = 0.46 mm^−^^1^
*c* = 29.7957 (9) Å	*T* = 200 K
β = 93.504 (3)°	Plate, pale yellow
*V* = 1453.16 (8) Å^3^	0.49 × 0.41 × 0.12 mm
*Z* = 4	

### Data collection

**Table:**

Oxford Diffraction Gemini R diffractometer	4842 independent reflections
Radiation source: fine-focus sealed tube	2832 reflections with *I* > 2σ(*I*)
graphite	*R*_int_ = 0.035
Detector resolution: 10.5081 pixels mm^-1^	θ_max_ = 32.6°, θ_min_ = 4.6°
φ and ω scans	*h* = −8→8
Absorption correction: multi-scan (*CrysAlis RED*; Oxford Diffraction, 2007)	*k* = −12→12
*T*_min_ = 0.887, *T*_max_ = 1.000	*l* = −44→44
13882 measured reflections	

### Refinement

**Table:**

Refinement on *F*^2^	Primary atom site location: structure-invariant direct methods
Least-squares matrix: full	Secondary atom site location: difference Fourier map
*R*[*F*^2^ > 2σ(*F*^2^)] = 0.038	Hydrogen site location: inferred from neighbouring sites
*wR*(*F*^2^) = 0.086	H-atom parameters constrained
*S* = 0.93	*w* = 1/[σ^2^(*F*_o_^2^) + (0.0416*P*)^2^] where *P* = (*F*_o_^2^ + 2*F*_c_^2^)/3
4842 reflections	(Δ/σ)_max_ = 0.001
208 parameters	Δρ_max_ = 0.29 e Å^−^^3^
0 restraints	Δρ_min_ = −0.36 e Å^−^^3^

### Special details

**Table:**

Experimental. (CrysAlis RED; Oxford Diffraction, 2007) Empirical absorption correction using spherical harmonics, implemented in SCALE3 ABSPACK scaling algorithm.
Geometry. All e.s.d.'s (except the e.s.d. in the dihedral angle between two l.s. planes) are estimated using the full covariance matrix. The cell e.s.d.'s are taken into account individually in the estimation of e.s.d.'s in distances, angles and torsion angles; correlations between e.s.d.'s in cell parameters are only used when they are defined by crystal symmetry. An approximate (isotropic) treatment of cell e.s.d.'s is used for estimating e.s.d.'s involving l.s. planes.
Refinement. Refinement of *F*^2^ against ALL reflections. The weighted *R*-factor *wR* and goodness of fit *S* are based on *F*^2^, conventional *R*-factors *R* are based on *F*, with *F* set to zero for negative *F*^2^. The threshold expression of *F*^2^ > σ(*F*^2^) is used only for calculating *R*-factors(gt) *etc*. and is not relevant to the choice of reflections for refinement. *R*-factors based on *F*^2^ are statistically about twice as large as those based on *F*, and *R*- factors based on ALL data will be even larger.

### Fractional atomic coordinates and isotropic or equivalent isotropic displacement parameters (Å^2^)

**Table:**

	*x*	*y*	*z*	*U*_iso_*/*U*_eq_	
Cl1	0.42210 (6)	0.13331 (4)	0.298854 (11)	0.02796 (10)	
Cl2	−0.42944 (7)	−0.05032 (6)	0.057164 (13)	0.04852 (14)	
O1	0.54644 (18)	0.07511 (14)	0.39281 (4)	0.0401 (3)	
O2	−0.26101 (17)	−0.20745 (14)	0.33569 (3)	0.0378 (3)	
O3	0.31583 (17)	−0.12832 (14)	0.24103 (3)	0.0364 (3)	
N	−0.02512 (19)	−0.06128 (15)	0.27365 (4)	0.0273 (3)	
H0A	−0.1786	−0.0415	0.2689	0.033*	
C1	0.3653 (2)	0.00688 (18)	0.38026 (5)	0.0275 (3)	
C2	0.2705 (2)	0.01309 (17)	0.33244 (4)	0.0251 (3)	
C3	0.0712 (2)	−0.06238 (17)	0.31773 (4)	0.0240 (3)	
C4	−0.0749 (2)	−0.14777 (18)	0.34975 (5)	0.0264 (3)	
C5	0.0144 (2)	−0.15604 (18)	0.39758 (5)	0.0272 (3)	
C6	−0.1138 (3)	−0.2369 (2)	0.42817 (5)	0.0391 (4)	
H6A	−0.2591	−0.2864	0.4186	0.047*	
C7	−0.0289 (3)	−0.2451 (3)	0.47288 (5)	0.0477 (5)	
H7A	−0.1169	−0.3002	0.4939	0.057*	
C8	0.1823 (3)	−0.1737 (2)	0.48705 (5)	0.0477 (5)	
H8A	0.2390	−0.1794	0.5177	0.057*	
C9	0.3111 (3)	−0.0940 (2)	0.45660 (5)	0.0391 (4)	
H9A	0.4574	−0.0459	0.4663	0.047*	
C10	0.2273 (2)	−0.08383 (18)	0.41170 (5)	0.0287 (3)	
C11	0.1070 (3)	−0.08964 (17)	0.23680 (5)	0.0263 (3)	
C12	−0.0252 (2)	−0.07098 (17)	0.19218 (4)	0.0245 (3)	
C13	−0.2316 (2)	0.01736 (18)	0.18635 (5)	0.0277 (3)	
H13A	−0.2882	0.0726	0.2110	0.033*	
C14	−0.3550 (3)	0.02492 (19)	0.14462 (5)	0.0315 (3)	
H14A	−0.4966	0.0846	0.1406	0.038*	
C15	−0.2697 (3)	−0.05531 (19)	0.10907 (5)	0.0307 (3)	
C16	−0.0608 (3)	−0.1395 (2)	0.11357 (5)	0.0319 (3)	
H16A	−0.0020	−0.1913	0.0885	0.038*	
C17	0.0618 (3)	−0.14706 (19)	0.15551 (5)	0.0295 (3)	
H17A	0.2058	−0.2045	0.1592	0.035*	

### Atomic displacement parameters (Å^2^)

**Table:**

	*U*^11^	*U*^22^	*U*^33^	*U*^12^	*U*^13^	*U*^23^
Cl1	0.02827 (17)	0.0286 (2)	0.02758 (17)	−0.00541 (15)	0.00608 (13)	0.00212 (15)
Cl2	0.0514 (2)	0.0680 (4)	0.02503 (18)	0.0176 (2)	−0.00744 (17)	−0.0029 (2)
O1	0.0364 (6)	0.0500 (8)	0.0334 (6)	−0.0141 (6)	−0.0034 (5)	−0.0042 (5)
O2	0.0321 (5)	0.0490 (8)	0.0318 (6)	−0.0152 (5)	−0.0010 (5)	0.0023 (5)
O3	0.0328 (6)	0.0476 (8)	0.0288 (5)	0.0120 (5)	0.0007 (4)	−0.0072 (5)
N	0.0240 (6)	0.0383 (8)	0.0196 (5)	0.0012 (5)	0.0019 (5)	0.0013 (5)
C1	0.0266 (7)	0.0307 (9)	0.0253 (7)	−0.0004 (6)	0.0021 (6)	−0.0031 (6)
C2	0.0272 (7)	0.0247 (8)	0.0239 (7)	0.0006 (6)	0.0058 (6)	−0.0012 (6)
C3	0.0252 (6)	0.0278 (8)	0.0192 (6)	0.0026 (6)	0.0028 (5)	−0.0013 (6)
C4	0.0278 (7)	0.0268 (8)	0.0248 (7)	−0.0014 (6)	0.0030 (6)	−0.0008 (6)
C5	0.0294 (7)	0.0302 (9)	0.0220 (6)	0.0000 (6)	0.0023 (6)	0.0005 (6)
C6	0.0374 (8)	0.0517 (12)	0.0286 (7)	−0.0087 (8)	0.0041 (7)	0.0049 (8)
C7	0.0502 (10)	0.0654 (14)	0.0282 (8)	−0.0078 (9)	0.0083 (7)	0.0117 (9)
C8	0.0532 (10)	0.0680 (15)	0.0214 (7)	0.0003 (10)	−0.0008 (7)	0.0049 (8)
C9	0.0374 (8)	0.0562 (12)	0.0232 (7)	−0.0047 (8)	−0.0026 (6)	−0.0018 (7)
C10	0.0296 (7)	0.0348 (9)	0.0218 (6)	0.0004 (6)	0.0023 (6)	−0.0028 (6)
C11	0.0306 (7)	0.0258 (8)	0.0228 (7)	0.0018 (6)	0.0033 (6)	−0.0012 (6)
C12	0.0275 (7)	0.0260 (8)	0.0201 (6)	−0.0020 (6)	0.0034 (5)	0.0002 (6)
C13	0.0321 (7)	0.0282 (8)	0.0233 (7)	0.0034 (6)	0.0060 (6)	−0.0017 (6)
C14	0.0293 (7)	0.0361 (9)	0.0291 (7)	0.0071 (7)	0.0022 (6)	0.0014 (7)
C15	0.0359 (8)	0.0359 (9)	0.0202 (6)	0.0015 (7)	−0.0008 (6)	0.0018 (7)
C16	0.0347 (8)	0.0400 (10)	0.0215 (7)	0.0064 (7)	0.0051 (6)	−0.0032 (7)
C17	0.0292 (7)	0.0348 (9)	0.0246 (7)	0.0052 (6)	0.0035 (6)	−0.0009 (6)

### Geometric parameters (Å, °)

**Table:**

Cl1—C2	1.7105 (15)	C7—C8	1.380 (2)
Cl2—C15	1.7394 (14)	C7—H7A	0.9500
O1—C1	1.2154 (17)	C8—C9	1.381 (2)
O2—C4	1.2166 (16)	C8—H8A	0.9500
O3—C11	1.2167 (16)	C9—C10	1.3932 (19)
N—C11	1.3834 (18)	C9—H9A	0.9500
N—C3	1.3890 (15)	C11—C12	1.4905 (19)
N—H0A	0.8800	C12—C17	1.392 (2)
C1—C10	1.480 (2)	C12—C13	1.392 (2)
C1—C2	1.4907 (18)	C13—C14	1.3867 (19)
C2—C3	1.3461 (19)	C13—H13A	0.9500
C3—C4	1.494 (2)	C14—C15	1.379 (2)
C4—C5	1.4829 (19)	C14—H14A	0.9500
C5—C6	1.387 (2)	C15—C16	1.381 (2)
C5—C10	1.391 (2)	C16—C17	1.3903 (19)
C6—C7	1.389 (2)	C16—H16A	0.9500
C6—H6A	0.9500	C17—H17A	0.9500
			
C11—N—C3	123.62 (11)	C8—C9—C10	120.27 (15)
C11—N—H0A	118.2	C8—C9—H9A	119.9
C3—N—H0A	118.2	C10—C9—H9A	119.9
O1—C1—C10	121.71 (13)	C5—C10—C9	119.60 (14)
O1—C1—C2	121.20 (14)	C5—C10—C1	121.47 (12)
C10—C1—C2	117.08 (12)	C9—C10—C1	118.91 (13)
C3—C2—C1	122.21 (13)	O3—C11—N	121.65 (12)
C3—C2—Cl1	122.74 (11)	O3—C11—C12	123.01 (13)
C1—C2—Cl1	114.90 (10)	N—C11—C12	115.34 (12)
C2—C3—N	124.82 (13)	C17—C12—C13	119.63 (12)
C2—C3—C4	120.79 (12)	C17—C12—C11	118.00 (12)
N—C3—C4	114.27 (12)	C13—C12—C11	122.36 (12)
O2—C4—C5	122.80 (13)	C14—C13—C12	120.19 (13)
O2—C4—C3	119.01 (12)	C14—C13—H13A	119.9
C5—C4—C3	118.19 (12)	C12—C13—H13A	119.9
C6—C5—C10	120.03 (13)	C15—C14—C13	119.16 (13)
C6—C5—C4	119.89 (13)	C15—C14—H14A	120.4
C10—C5—C4	120.08 (13)	C13—C14—H14A	120.4
C5—C6—C7	119.71 (15)	C14—C15—C16	121.78 (13)
C5—C6—H6A	120.1	C14—C15—Cl2	119.11 (11)
C7—C6—H6A	120.1	C16—C15—Cl2	119.11 (11)
C8—C7—C6	120.49 (16)	C15—C16—C17	118.79 (14)
C8—C7—H7A	119.8	C15—C16—H16A	120.6
C6—C7—H7A	119.8	C17—C16—H16A	120.6
C7—C8—C9	119.89 (14)	C16—C17—C12	120.37 (13)
C7—C8—H8A	120.1	C16—C17—H17A	119.8
C9—C8—H8A	120.1	C12—C17—H17A	119.8
			
O1—C1—C2—C3	179.54 (14)	C6—C5—C10—C1	−178.31 (15)
C10—C1—C2—C3	−1.3 (2)	C4—C5—C10—C1	2.1 (2)
O1—C1—C2—Cl1	−4.8 (2)	C8—C9—C10—C5	−0.8 (3)
C10—C1—C2—Cl1	174.35 (11)	C8—C9—C10—C1	177.93 (16)
C1—C2—C3—N	−179.82 (14)	O1—C1—C10—C5	177.09 (15)
Cl1—C2—C3—N	4.8 (2)	C2—C1—C10—C5	−2.0 (2)
C1—C2—C3—C4	4.4 (2)	O1—C1—C10—C9	−1.6 (2)
Cl1—C2—C3—C4	−170.99 (11)	C2—C1—C10—C9	179.29 (14)
C11—N—C3—C2	49.6 (2)	C3—N—C11—O3	5.2 (2)
C11—N—C3—C4	−134.37 (14)	C3—N—C11—C12	−175.37 (13)
C2—C3—C4—O2	175.91 (14)	O3—C11—C12—C17	21.9 (2)
N—C3—C4—O2	−0.3 (2)	N—C11—C12—C17	−157.54 (14)
C2—C3—C4—C5	−4.1 (2)	O3—C11—C12—C13	−159.00 (15)
N—C3—C4—C5	179.64 (12)	N—C11—C12—C13	21.6 (2)
O2—C4—C5—C6	1.2 (2)	C17—C12—C13—C14	2.4 (2)
C3—C4—C5—C6	−178.73 (15)	C11—C12—C13—C14	−176.72 (14)
O2—C4—C5—C10	−179.23 (15)	C12—C13—C14—C15	−0.4 (2)
C3—C4—C5—C10	0.8 (2)	C13—C14—C15—C16	−1.9 (2)
C10—C5—C6—C7	0.1 (3)	C13—C14—C15—Cl2	178.10 (12)
C4—C5—C6—C7	179.64 (16)	C14—C15—C16—C17	2.1 (2)
C5—C6—C7—C8	−0.2 (3)	Cl2—C15—C16—C17	−177.92 (13)
C6—C7—C8—C9	−0.3 (3)	C15—C16—C17—C12	0.0 (2)
C7—C8—C9—C10	0.7 (3)	C13—C12—C17—C16	−2.2 (2)
C6—C5—C10—C9	0.4 (2)	C11—C12—C17—C16	176.94 (14)
C4—C5—C10—C9	−179.17 (15)		

### Hydrogen-bond geometry (Å, °)

**Table:**

*D*—H···*A*	*D*—H	H···*A*	*D*···*A*	*D*—H···*A*
N—H0A···Cl1^i^	0.88	2.89	3.6491 (12)	145
C14—H14A···O2^ii^	0.95	2.40	3.2517 (19)	149

Symmetry codes: (i) *x*−1, *y*, *z*; (ii) −*x*−1, *y*+1/2, −*z*+1/2.
